# Deep Sequencing Analysis Reveals the Mycoviral Diversity of the Virome of an Avirulent Isolate of *Rhizoctonia solani* AG-2-2 IV

**DOI:** 10.1371/journal.pone.0165965

**Published:** 2016-11-04

**Authors:** Anika Bartholomäus, Daniel Wibberg, Anika Winkler, Alfred Pühler, Andreas Schlüter, Mark Varrelmann

**Affiliations:** 1 Institute of Sugar Beet Research, D-37077, Göttingen, Germany; 2 Institute for Genome Research and Systems Biology, CeBiTec, Bielefeld University, D-33501, Bielefeld, Germany; National University of Ireland—Galway, IRELAND

## Abstract

*Rhizoctonia solani* represents an important plant pathogenic *Basidiomycota* species complex and the host of many different mycoviruses, as indicated by frequent detection of dsRNA elements in natural populations of the fungus. To date, eight different mycoviruses have been characterized in *Rhizoctonia* and some of them have been reported to modulate its virulence. DsRNA extracts of the avirulent *R*. *solani* isolate DC17 (AG2-2-IV) displayed a diverse pattern, indicating multiple infections with mycoviruses. Deep sequencing analysis of the dsRNA extract, converted to cDNA, revealed that this isolate harbors at least 17 different mycovirus species. Based on the alignment of the conserved RNA-dependent RNA-polymerase (RdRp) domain, this viral community included putative members of the families *Narnaviridae*, *Endornaviridae*, *Partitiviridae* and *Megabirnaviridae* as well as of the order *Tymovirales*. Furthermore, viruses, which could not be assigned to any existing family or order, but showed similarities to so far unassigned species like *Sclerotinia sclerotiorum RNA virus L*, *Rhizoctonia solani dsRNA virus 1*, *Aspergillus foetidus slow virus 2* or *Rhizoctonia fumigata virus 1*, were identified. This is the first report of a fungal isolate infected by 17 different viral species and a valuable study case to explore the diversity of mycoviruses infecting *R*. *solani*.

## Introduction

*Rhizoctonia solani* Kühn [teleomorph: *Thanatephorus cucumeris* (Frank) Donk] represents an important species complex of known plant pathogens, which infect a wide variety of plant species including many economically important crops [1]. Due to its soil-borne nature, facultative saprophytism and the wide host-range, this pathogen is difficult to control by conventional agronomic measures like fungicides, crop rotation or plant resistance [2]. *Rhizoctonia* is considered as ubiquitous in soil, but has limited abilities of long distance spread, because it does not produce asexual spores [1, 3]. To understand the epidemiology of diseases caused by *Rhizoctonia*, especially regarding the disease suppression observed in association with monoculture of host plants, the interaction of the fungus with its environment and, in particular, the microflora has been studied by many researchers [4]. Today, it is well established that *Rhizoctonia* is significantly affected by suppressive organisms, but the nature of this biocontrol is still a matter of debate [4]. The finding that hypovirulent isolates of *Rhizoctonia* can be successfully used for biocontrol purposes, raised also interest in the possible involvement of mycoviruses [5, 6].

Mycoviruses have been identified in all major fungal taxa and the number of newly described species has expanded enormously during the last years [7]. The increasing usage of deep sequencing approaches will further enhance this trend, leading to a better understanding of the diversity of mycoviruses and the interaction with their hosts. To date, thirteen viral families have been recognized by the International Committee for the Taxonomy of Viruses (ICTV) that harbor mycoviruses [8]. Most of the mycoviruses characterized possess an RNA genome consisting either of single-stranded or double-stranded RNA, which in some cases is segmented [7]. In general, mycoviruses are considered as endogenous viral elements without an extracellular phase. An exception from this assumption has been discovered recently by Yu and coworkers, who identified a novel mycovirus species, related to the ssDNA *Geminiviridae*, which is reported to be transmitted via spray application of purified virions and induces hypovirulence [9]. Nevertheless, in most cases mycoviral infections are cryptic [10, 11] and only little is known about the interaction of these pathogens with their hosts [12]. The advances in the field of mycovirology achieved during the last years indicate that these interactions are rather diverse, ranging from mutualism, e.g. improvement of the adaption abilities of the host towards abiotic stress [13], to severe impairment including irregular growth, diminished reproduction, lack of pigmentation and hypovirulence [14]. Using mycoviruses, which induce such impairments in their host, for the control of plant pathogens in agricultural systems is a tempting approach. Factors, like the homogenous environmental conditions and the low species diversity, which facilitate the development of plant diseases, might also facilitate the spread and development of mycoviral infections of the plant pathogens [15]. Successful examples, like the biocontrol of chestnut blight (*Cryphonectria parasitica*) caused by hypoviruses, raises hope to exploit also other mycoviruses as biocontrol agents [16]. Nevertheless, the first step towards a mycovirus based biocontrol system is the identification of suitable viral candidates. Numerous studies have been carried out indicating a high diversity among *R*. *solani* infecting mycoviruses [17–20]. However, only a small number of different species has been characterized so far, including species of the *Partitiviridae*, *Endornaviridae*, *Narnaviridae* and a few unclassified mycoviruses [21–28]. Deep sequencing analysis, especially of viral metagenomes, is currently the most efficient way to identify unknown viruses. On the one hand, it might allow the identification of promising biocontrol candidates and on the other hand, it can provide important information about the global ecology and evolution of mycoviruses [15, 29]. Until now, studies showed that 60 to 95% of the sequences derived from viral metagenome sequencing do not find homologous sequences in the NCBI database and mycoviruses are less well studied compared to animal or plant viruses [12]. This leads to significant difficulties in the characterization of novel mycoviruses from complex viromes. The RNA-dependent RNA polymerase (RdRp) gene is the only gene that is universally conserved among RNA viruses and represents the key for the identification of evolutionary relationships between the respective viruses [30–32]. The RdRp domain is typically smaller than 400 amino acids in length. Apart from seven conserved motifs, named G, F1-3, A, B, C, D, E, which are essential for its function and represent about 75% of the domain, the RdRp sequences are highly variable [33]. Considering the diversity of RNA viruses, the sequence similarity of the RdRp domain is too low for the construction of evolutionary trees of all viral families, as only a few amino acid residues like a conserved aspartic acid in motif A exist in all viruses [33, 34]. However, the analysis of the conserved RdRp domain allows the identification of evolutionary lineages, named superfamilies, which show a higher level of conservation within this group [33, 35]. Furthermore, phylogenetic analysis of the conserved RdRp domain within these superfamilies enables a classification of RNA viruses on family level. The identification of novel RdRp genes in viral genomes therefore represents an efficient way to estimate the viral diversity in the sample, making first taxonomic assignments possible and permits the fast identification of unknown viruses. This study describes the diversity of a fungal virome consisting of a high number of novel RNA mycoviruses, using the viral hallmark gene RdRp for classification.

## Material & Methods

### Fungal isolate and culturing

The fungal strain DC17, originally isolated from sugar beet, was derived from the IRS (Institute of Sugar Beet Research, Netherlands) and identified as anastomosis group 2-2IV by sequencing of the internal transcribed spacer region [36]. The isolate showed unusual light pigmentation, slow growth on PDA and hypovirulence in a damping-off assay with sugar beet (data not shown). The strain was stored at -80°C on PDA. For extraction of DNA, RNA and dsRNA, mycelium was grown in PDB at 24°C.

### Extraction of dsRNA from fungal mycelium

The dsRNA was extracted using a modified version of the protocol published by Valverde and colleagues [37]. Mycelium (10.3 g) was homogenized in liquid nitrogen in the presence of 0.25 g of polyvinylpolypyrrolidone. The pulverized mycelium was suspended in 10 mL 2xSTE buffer (0.1 M Tris-HCl, pH 6.8, 2 mM EDTA, 0.2 M NaCl_2_) containing 0.25 g polyvinylpyrrolidone. Nucleic acids were extracted by addition of 2 mL beta-mercaptoethanol, 0.5 mL SDS (20%), 20 mL phenol/chloroform (1:1) and the suspension was incubated on a shaker for 30 min at RT, followed by centrifugation for 20 min at 5000 xg. The aqueous phase was supplemented with pure ethanol to a final concentration of 16%, mixed with 2 g CF-11 cellulose (Whatman) and incubated for another 20 min at RT. Contaminants were removed by three washing steps with STE16 (STE + 16% EtOH) followed by centrifugation for 2 min at 500 xg. The cellulose was resuspended in 20 mL STE16 and transferred into Econo-Columns (Biorad). After an additional washing step with 20 mL STE16, the dsRNA was eluted by the addition of 10 mL 2xSTE. To remove contaminating genomic DNA and RNA, the eluate was treated with T1-RNase (Roche) (1 U/mL) (30 min, 37°C) followed by the addition of 3.3% 1 M MgCl_2_ and a treatment with DNase I (Roche) (2 U/mL) for 30 minutes at 37°C each. Thereafter the extract was supplemented with EtOH to a final concentration of 16% and 3.3% 0.5 M EDTA was added. The eluate was mixed with 2 g cellulose and treated as described above. The dsRNA was eluted with 8 mL STE. For precipitation, 80% isopropanol was added. The mixture was incubated at -20°C over night and centrifuged for 30 min at 10 000 xg. After a washing step with 70% EtOH, the dsRNA was resolved in TE buffer and checked by gel electrophoresis for quantity and quality control.

### cDNA synthesis and library preparation for high-throughput sequencing

The cDNA library was prepared following a modified version of the protocol of Froussard [38]. Four μL of dsRNA were mixed with 100 pmol universal Primer-dN6 (GCCGGAGCTCTGCAGAATTCNNNNNN), incubated for 2 min at 99°C and chilled on ice. An enzyme mix was prepared using 6.25 mM dNTPs, 50 U RevertAid H-Minus Reverse transcriptase (Thermo Scientific) and 5 μL RT buffer in a volume of 20 μL and added to the dsRNA for an incubation of 30 min at 42°C. The cDNA was purified using the NucleoSpin, PCR clean-up, gel extraction kit (Macherey-Nagel) following the manufacturer’s instructions. The purified cDNA (25 μL) was mixed with 100 pmol of Primer-dN6, incubated for 2 min at 99°C and chilled on ice. For second strand synthesis, a mixture containing 3.125 mM dNTPs, 5 U Klenow-fragment (Thermo Scientific) and 5 μL of 10x Klenow-buffer in a total volume of 24 μL was added and incubated at 37°C for 30 min. The generated dscDNA was again purified and 5 μL were used for amplification. The PCR reaction mixture contained 125 nmol MgCl_2_, 0.15 pmol Primer (GCCGGAGCTCTGCAGAATTC), 12.5 nmol dNTPs, 1 U Dreamtaq (Thermo Scientific) and 5 μL 10x Dreamtaq buffer in a total volume of 50 μL. PCR conditions were as follows: Initial denaturation at 94°C for 1 min, followed by 30 cycles of 20 sec 94°C, 30 seconds 55°C, 2 min 72°C and a final elongation step of 10 min at 72°C. PCR products were analyzed by gel electrophoresis and fragments between 0.5 and 2 kb were excised and extracted from the gel using the NucleoSpin, PCR clean-up, gel extraction kit. Fragments were sheared and adaptors for Illumina sequencing were ligated [39].

### High-throughput sequencing on the Illumina MiSeq system and sequence analysis

DNA quality was assessed by gel-electrophoresis and the quantity was estimated by a fluorescence-based method using the Quant-iT PicoGreen dsDNA kit (Invitrogen) and the Tecan Infinite 200 Microplate Reader (Tecan Deutschland GmbH). The *R*. *solani* DC17 virome was established on the Illumina MiSeq system by a pair-end sequencing run (2 × 300 bp) with a distance range of about 500 bp. A *de novo* assembly was performed using the gsAssembler software (version 2.8.) with default settings. Resulting virus sequences were *in silico* finished [40], imported into the automatic annotation platform GenDB [41] and refined manually. Raw data was deposited the European Nucleotide Archive (ENA) under the accession number PRJEB15275. In addition, all generated contigs were checked for open reading frames using the ORF finder software at the NCBI website (http://www.ncbi.nlm.nih.gov/gorf/gorf.html). Predicted amino acid sequences were subsequently compared to the NCBI database using BLASTP with standard settings and conserved domains were identified using the conserved domain database at the NCBI website (http://www.ncbi.nlm.nih.gov/cdd).

### Completion of partial RdRp domains

To complete partial RdRp domains derived from smaller contigs, cDNA was synthesized using a sequence specific primer based on known sequence information targeting the whole RdRp domain (S1 Table). cDNA synthesis followed by dscDNA synthesis was performed as described above. After PCR amplification the resulting fragments were gel purified, fragments between 800 und 2000 bp were excised and extracted. The fragments were cloned using the pJet1.2 cloning Kit (Fermentas) following the manufacturer’s instructions. The resulting clones were sequenced with specific primers by standard Sanger-sequencing (Seqlab).

### Confirmation of sequence assembly results

Specific primers were designed according to the results of the Illumina sequencing, flanking the region of the conserved RdRp domains. Primer sequences are listed in S1 Table. For cDNA synthesis total RNA was extracted using the RNeasy Plant Mini kit (Qiagen) according to the manufacturer’s instructions. cDNA synthesis and PCR amplification were done as described above using 2 μL cDNA. PCR products were gel purified and sequenced.

### Identification of conserved sequence motifs and phylogenetic analysis

Nucleotide sequences, which were confirmed by resequencing with specific primers, were translated into amino acid sequences and aligned to reference genomes from the NCBI database. Conserved motifs were identified by comparisons with well characterized RdRps based on work published by Bruenn 2003, Černý et al. 2014, Koonin et al. 1993, Boehr et al. 2014 and Xu et al. 2003 [30, 42–44]. For phylogenetic analysis, the RdRp domains, spanning the identified conserved motifs, were aligned in MEGA 6.06 using MUSCLE with standard settings and Neighbor-joining trees were constructed with 1000 bootstrap replicates [45]. Sequences spanning the conserved motifs A to G were deposited in the NCBI GenBank database under the accession numbers KX291005 and KX349056—KX349071.

## Results and Discussion

### Virome sequencing of *R*. *solani* isolate DC17

**Fig 1:**
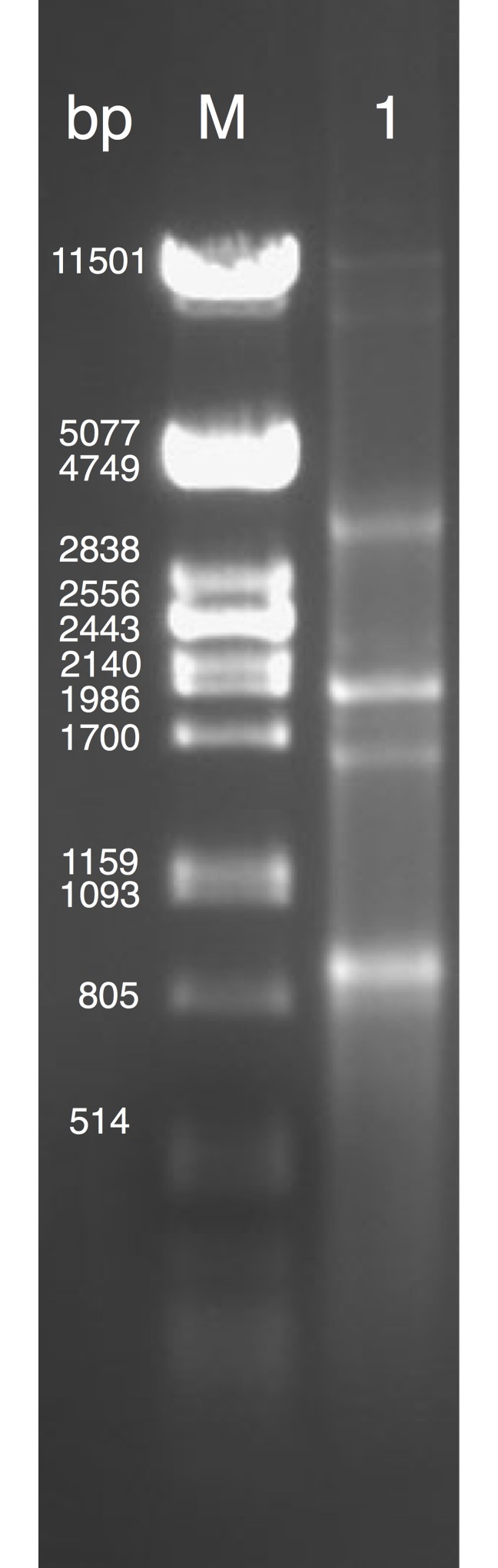
Purified dsRNA extracted from *R*. *solani* DC17. Electrophoresis of dsRNA extract from DC17 (1) in a 1% agarose gel stained with ethidium bromide showing several dsRNA fragments ranging from approx. 0.85 to 12 kb in size compared to a size marker of a *Pst*I digested lambda DNA (M).

The dsRNA extract of the *R*. *solani* isolate DC17 showed a diverse pattern of dsRNA fragments after gel electrophoresis indicating the presence of multiple mycoviruses. At least six dsRNA fragments with a mobility corresponding to the sizes of approximately 0.85 to 12 kb were identified (Fig 1).

Sequencing on the Illumina MiSeq System resulted in a total of 930,558 paired-end reads yielding 279 Mb sequence information. After quality analysis, 0.1% of these reads were excluded from further analysis. Based on at least 90% sequence identity over a minimum of 40 bases, reads were assembled into 144 contigs with sizes between 12.8 and 0.4 kb. In total, 40.2% of the contigs were classified as viral sequences based on a BLAST search corresponding to about 84% of the used reads. The average read coverage of the contigs was approx. 270-fold, but great differences between contigs were observed (~15x–4500x). Based on the assembled virome data, it was possible to identify 17 contigs encoding different viral RdRp domains. For detailed analysis, these RdRp domains were re-sequenced based on whole RNA extracts and partial domains were completed. These data were used for taxonomic classification. The identified RdRps belong to four different protein families (Pfam) corresponding to either the alphavirus-like superfamily, the picornavirus-like super family or the *Narnaviridae*. They showed similarities from 31 to 93% to RdRp domains of viral sequences deposited in the NCBI database.

### Putative members of the family *Narnaviridae* identified in *R*. *solani* DC17

**Fig 2:**
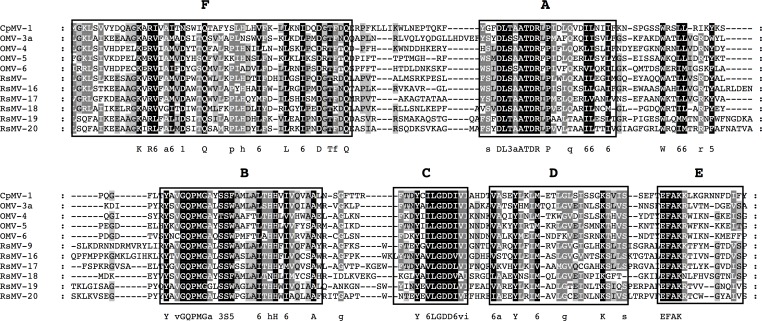
Identification of the conserved motifs A to F in the RdRp domain of the genus *Mitovirus*. The RdRp domains of putative mitoviruses identified in *R*. *solani* DC17 and recognized members of the genus mitovirus [8] were aligned using MEGA 6.06 and the sequence alignment algorithm MUSCLE [47]. Conserved motifs A-F were marked according to Bruenn 2003, Černý et al. 2014, Koonin et al. 1993, Boehr et al. 2014 and Xu et al. 2003 [32, 36, 44–46]. CpMV-1, *Cryphonectria parasitica mitovirus* (NP_660174.1); OMV-3a, *Ophiostoma mitovirus 3a* (CAJ32468.1); OMV-4, *Ophiostoma mitovirus 4* (NP_660179.1); OMV-5, *Ophiostoma mitovirus 5* (NP_660180.1); OMV-6, *Ophiostoma mitovirus 6* (NP_660181.1), RsMV-9, *Rhizoctonia solani mitovirus 9* DC17 (KX349058); RsMV-16, *Rhizoctonia solani mitovirus 16* (KX349057); RsMV-17, *Rhizoctonia solani mitovirus 17* (KX349059); RsMV-18, *Rhizoctonia solani mitovirus 18* (KX349060); RsMV-19, *Rhizoctonia solani mitovirus 19* (KX349056); RsMV-20, *Rhizoctonia solani mitovirus 20* (KX291005). Shading indicates level of conservation and the consensus sequence is displayed.

**Fig 3:**
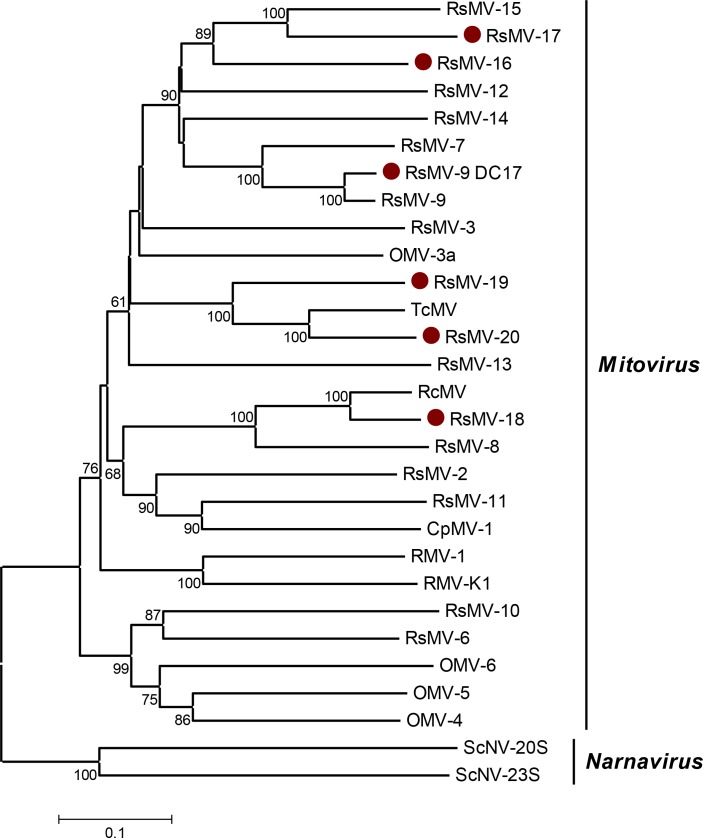
Phylogenetic analysis of the members of the family *Narnaviridae*. An unrooted phylogenetic tree of the *Narnaviridae* was calculated based on the alignment of the RdRp domain spanning the conserved motifs A to F using the neighbor-joining method with 1000 bootstrap replicates. The scale represents a genetic distance of 0.1 amino acid substitutions per site. Bootstrap values < 50% are not shown. Analyzed species include mitoviruses identified in *Rhizoctonia* as well as species of the family *Narnaviridae* recognized by the ICTV. Species identified in this study are marked with dots. Analysis was conducted using MEGA 6.06 and the sequence alignment algorithm MUSCLE [45]. CpMV-1, *Cryphonectria parasitica mitovirus* (NP_660174.1); OMV-3a, *Ophiostoma mitovirus 3a* (CAJ32468.1); OMV-4, *Ophiostoma mitovirus 4* (NP_660179.1); OMV-5, *Ophiostoma mitovirus 5* (NP_660180.1); OMV-6, *Ophiostoma mitovirus 6* (NP_660181.1); ScNV-20S, *Saccharomyces 20S RNA narnavirus* (NP_660178.1); ScNV-23S, *Saccharomyces 23S RNA narnavirus* (NP_660177.1); RsMV-1, *Rhizoctonia solani mitovirus 1* (ALD89121.1); TcMV-1, *Thanatephorus cucumeris mitovirus 1* (AAD17381.1); RsMV-2, *Rhizoctonia solani mitovirus 2* (ALD89121.1); RsMV-3, *Rhizoctonia solani mitovirus 3* (ALD89122.1); RsMV-4, *Rhizoctonia solani mitovirus 4* (ALD89123.1); RsMV-5, *Rhizoctonia solani mitovirus 5* (ALD89124.1); RsMV-6, *Rhizoctonia solani mitovirus 6* (ALD89125.1); RsMV-7, *Rhizoctonia solani mitovirus 7* (ALD89126.1); RsMV-8, *Rhizoctonia solani mitovirus 8* (ALD89127.1); RsMV-9, *Rhizoctonia solani mitovirus 9* (ALD89128.1); RsMV-10, *Rhizoctonia solani mitovirus 10* (ALD89102.1); RsMV-11, *Rhizoctonia solani mitovirus 11* (ALD89116.1); RsMV-12, *Rhizoctonia solani mitovirus 12* (ALD89117.1); RsMV-13, *Rhizoctonia solani mitovirus 1*3 (ALD89118.1); RsMV-14, *Rhizoctonia solani mitovirus 14* (ALD89119.1); RsMV-15, *Rhizoctonia solani mitovirus 15* (ALD89120.1); RsMV-K1, *Rhizoctonia solani mitovirus K1* (ALD60243.1); RcMV, *Rhizoctonia cerealis mitovirus* (AIT71973.1); RsMV-9, *Rhizoctonia solani mitovirus 9* DC17 (KX349058); RsMV-16, *Rhizoctonia solani mitovirus 16* (KX349057); RsMV-17, *Rhizoctonia solani mitovirus 17* (KX349059); RsMV-18, *Rhizoctonia solani mitovirus 18* (KX349060); RsMV-19, *Rhizoctonia solani mitovirus 19* (KX349056); RsMV-20, *Rhizoctonia solani mitovirus 20* (KX349062).

Viruses of the family *Narnaviridae* are the simplest of all RNA viruses consisting only of one small single-stranded RNA of 2.3 to 3.6 kb, which codes for a single RdRp gene [46]. The family is divided into the two genera *Narnavirus* and *Mitovirus*. The members of the *Narnaviridae* are only distantly related to other RNA viruses infecting eukaryotes and share higher sequence similarity to bacteriophages of the family *Leviviridae* [47]. The analysis of the conserved RdRp domains revealed that the *R*. *solani* isolate DC17 is the host of six different mitoviruses. Sequence comparisons with all recognized members of the genus *Mitovirus* allowed the identification of the four conserved motifs I to IV previously described for mitoviruses by Kitahara and coworkers [48]. Multiple alignments identified these motifs as motif F to C and, additionally, motif E and D were detected in this study (Fig 2). So far, 18 different mitoviruses have been characterized in the genus *Rhizoctonia*, the majority of them in *R*. *solani* [49, 50]. Analysis of the conserved RdRp domain indicated that five of the mitoviruses found in this study, represent novel species, as they display only limited sequence similarity to the mitoviruses described so far (Fig 3). These mitoviruses were named *Rhizoctonia solani mitovirus 16* to *20* (RsMV-16 to 20). The RdRp domain of one mitovirus identified in the analyzed virome shows high sequence similarity of 92% to *Rhizoctonia solani mitovirus 9* (KP900918.1). Since only a partial sequence of this virus has been deposited in the NCBI database, no comparison of the complete RdRp protein was possible, to check whether both viruses fulfill the species demarcation criterion of less than 90% amino acid sequence similarity over the complete RdRp protein [8]. This mitovirus was presumably considered as *Rhizoctonia solani mitovirus 9* DC17.

### Putative member of the family *Endornaviridae* identified in *R*. *solani* DC17

**Fig 4:**
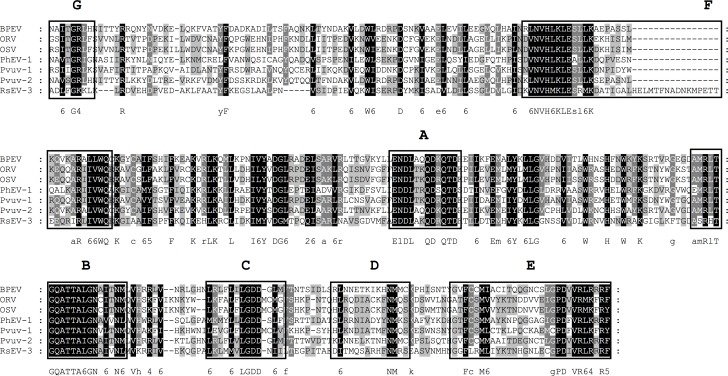
Identification of the conserved motifs A to G in the RdRp domain of the family *Endornaviridae*. The RdRp domains of the recognized members of the family *Endornaviridae* [8] and *Rhizoctonia solani endornavirus 3* (RsEV-3) (KX349065) were aligned using MEGA 6.06 and the sequence alignment algorithm MUSCLE [45]. Conserved motifs A-G were marked according to Bruenn 2003, Černý et al. 2014, Koonin et al. 1993, Boehr et al. 2014 and Xu et al. 2003 [32, 36, 44–46]. BPEV, *Bell pepper endornavirus* (AKP92841.1); ORV, *Oryza rufipogon endornavirus* (YP_438202.1); OSV, *Oryza sativa endornavirus* (YP_438200.1); PhEV-1, *Phytophthora endornavirus 1* (YP_241110.1); Pvuv-1, *Phaseolus vulgaris endornavirus 1* (YP_009011062.1); Pvuv-1, *Phaseolus vulgaris endornavirus 2* (ALJ56098.1). Shading indicates level of conservation and the consensus sequence is displayed.

Viruses belonging to the family *Endornaviridae* are found in algae, plants and fungi [8]. They possess a non-segmented genome of 14 to 17 kb which codes for a single large polyprotein [7]. The polyprotein always carries a conserved RdRp domain (pfam00978), specifying its affiliation to the alphavirus-like superfamily. Other typical conserved replicase motifs like a methyltransferase, helicase or glycosyltransferase are missing in some species [51]. The CDD search of the deduced gene products derived from the deep sequencing analysis of dsRNA revealed the presence of one conserved RdRp domain belonging to the pfam00978 family. It is 45% identical to the conserved RdRp domain of *Soybean-associated endornavirus 1*, which is the species in the NCBI database showing the highest level of similarity [52]. Seven conserved motifs (A-G), typical for members of the family *Endornaviridae* were identified by sequence alignments with all recognized members of this family (Fig 4). This indicates the presence of a novel endornavirus, named *Rhizoctonia solani endornavirus 3* (RsEV-3).

This finding was confirmed by a phylogenetic analysis of the conserved RdRp domain within the alphavirus-like superfamily which placed this novel virus within the family *Endornaviridae*. So far, three mycoviruses belonging to the family of the *Endornaviridae* were described to infect the genus *Rhizoctonia*, namely *Rhizoctonia solani endornavirus—RS002* (RsEV-1), *Rhizoctonia solani endornavirus 2* (RsEV-2) and *Rhizoctonia cerealis endornavirus 1* (RcEV-1) [28,50, 53]. Sequence comparisons showed that RsEV-3 and RsEV-1 possess 44.2% amino acid homology across the conserved RdRp domain. Comparison of the conserved motifs revealed that the RdRp domain of RsEV1 is incomplete, as the motifs C to E were missing. Sequence comparison of the remaining RdRp domain shows a homology of 52.5% to RsEV-1. Recently, the genome of RsEV-2 was deposited in the NCBI database [50]. Both viruses share 63.2% sequence identity within the RDRP domain. Due to the high conservation within this region, similarities between different members of the *Endornaviridae* can also be high, like it is the case for the *Bell pepper endornavirus* and *Phaseoulus vulgaris endornavirus*, which share a sequence homology of 74% within their RdRp domains.

### Putative members of the order *Tymovirales* identified in *R*. *solani* DC17

**Fig 5:**
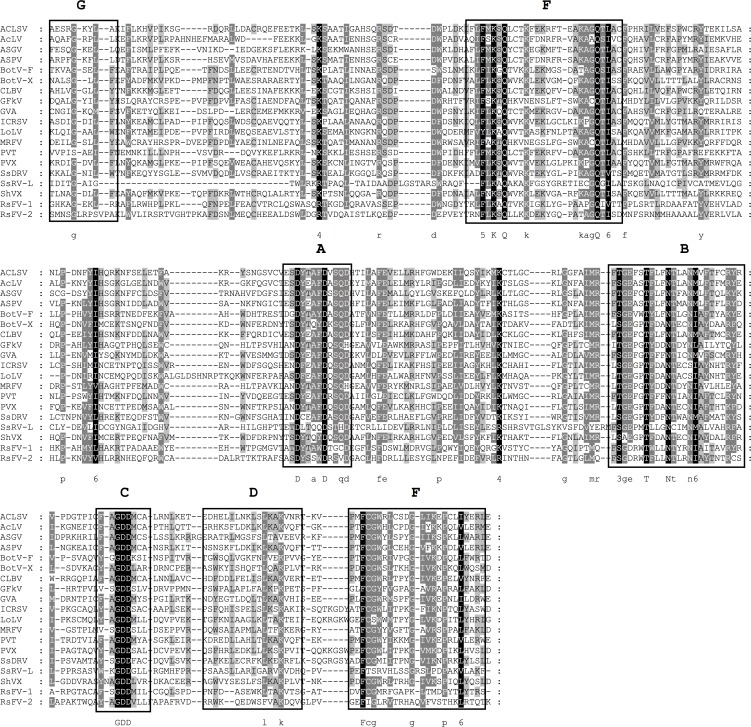
Identification of the conserved motifs A to G in the RdRp domain of the type species of all genera within the order *Tymovirales*. The RdRp domains of *Rhizoctonia solani flexivirus 1* (RsFV-1) (KX349064), *Rhizoctonia solani flexivirus 2* (RSFV-2) (KX349069) and the type species of all genera within the *Tymovirales* [8] were aligned using MEGA 6.06 and the sequence alignment algorithm MUSCLE [44]. Conserved motifs A to G were marked according to Bruenn 2003, Černý et al. 2014, Koonin et al. 1993, Boehr et al. 2014 and Xu et al. 2003 [30, 34, 42–44]. ACLSV, *Apple chlorotic leaf spot virus* (ABY71563.1); AcLV, *Aconitum latent virus* (NP_116487.1); ASGV, *Apple stem grooving virus* (BAA98054.1); ASPV, *Apple stem pitting virus* (AEP02955.1); BotV-F, *Botrytis virus F* (NP_068549.1); BotV-X, *Botrytis virus X (*NP_932306.1); CLBV, *Citrus leaf blotch virus* (NP_624333.1); GFkV, *Grapevine fleck virus* (NP_542612.1); GVA, *Grapevine virus A* (AAO17778.1); ICRSV, *Indian citrus ringspot virus* (NP_203553.1); LoLV, *Lolium latent virus* (YP_001718499.1); MRFV, *Maize rayado fino virus* (NP_115454.1); PVT, *Potato virus T* (BAM16482.1); PVX, *Potato virus X* (AAF89747.1); SsDRV, *Sclerotinia sclerotiorum debilitation-associated RNA virus* (YP_325662.1); ShVX, *Shallot virus X* (NP_620648.1). Shading indicates level of conservation and the consensus sequence is displayed.

**Fig 6:**
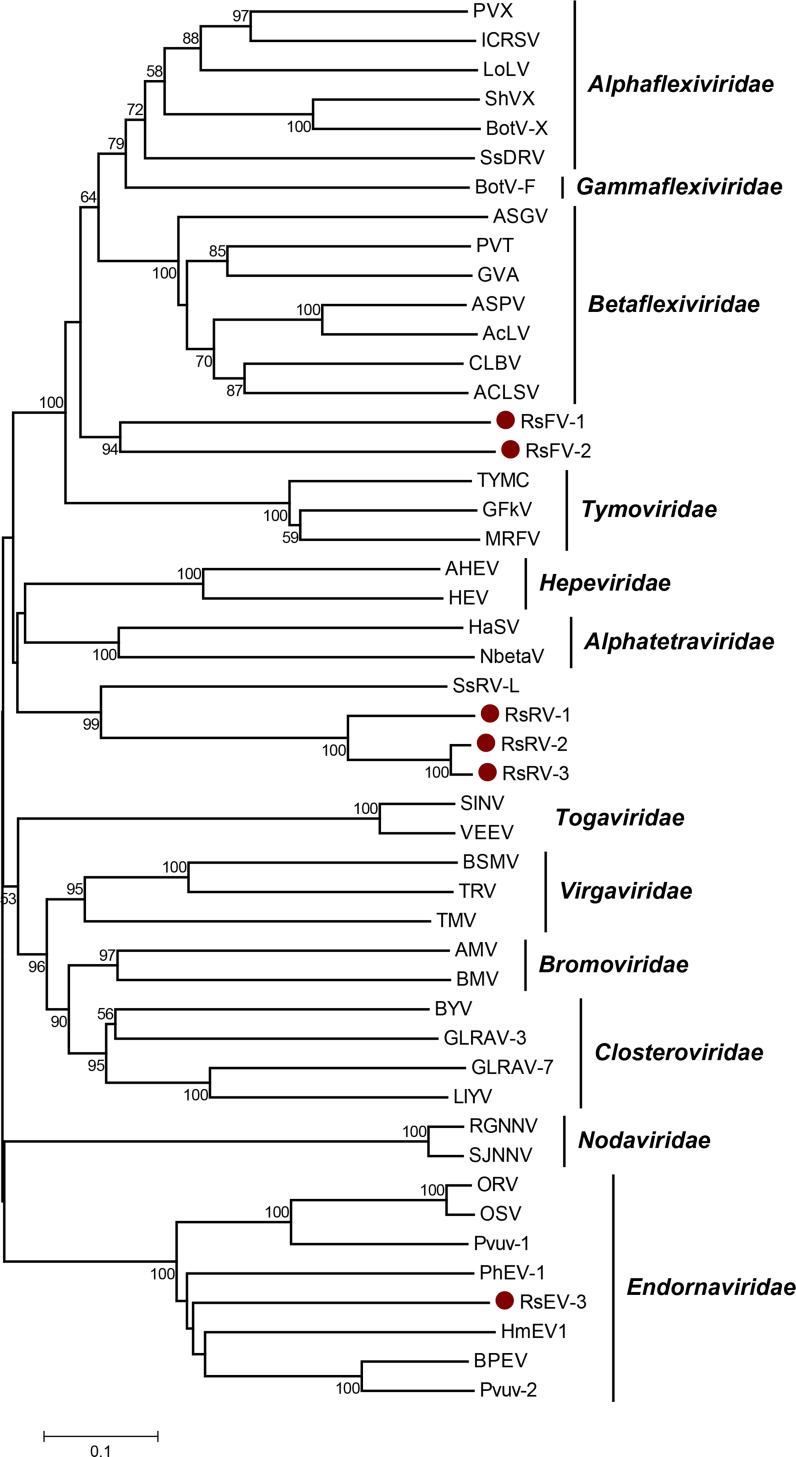
Phylogenetic analysis of selected type species within the families of the Alphavirus-like superfamily. An unrooted phylogenetic tree of the alphavirus-like superfamily was calculated based on the alignment of the RdRp domain spanning the conserved motifs A to G using the neighbor-joining method with 1000 bootstrap replicates. The scale represents a genetic distance of 0.1 amino acid substitutions per site. Bootstrap values < 50% are not shown. Newly identified species are marked with dots and compared to their next relatives and selected type species within families proposed to form the alphavirus-like superfamily [8, 29]. Analysis was conducted using MEGA 6.06 and the sequence alignment algorithm MUSCLE [45]. BPEV, *Bell pepper endornavirus* (AKP92841.1); ORV, *Oryza rufipogon endornavirus* (YP_438202.1); OSV, *Oryza sativa endornavirus* (YP_438200.1); PhEV-1, *Phytophthora endornavirus 1* (YP_241110.1); Pvuv-1, *Phaseolus vulgaris endornavirus 1* (YP_009011062.1); Pvuv-1, *Phaseolus vulgaris endornavirus 2 (*ALJ56098.1); SsRV-L, *Sclerotinia sclerotiorum RNA virus L* (ACE88957.1); ACLSV, *Apple chlorotic leaf spot virus* (ABY71563.1); AcLV, *Aconitum latent virus* (NP_116487.1); ASGV, *Apple stem grooving virus* (BAA98054.1); ASPV, *Apple stem pitting virus* (AEP02955.1); BotV-F, *Botrytis virus F* (NP_068549.1); BotV-X, *Botrytis virus X* (NP_932306.1); CLBV, *Citrus leaf blotch virus* (NP_624333.1); GFkV, *Grapevine fleck virus* (NP_542612.1); GVA, *Grapevine virus A* (AAO17778.1); ICRSV, *Indian citrus ringspot virus* (NP_203553.1); LoLV, *Lolium latent virus* (YP_001718499.1); MRFV, *Maize rayado fino virus* (NP_115454.1); PVT, *Potato virus T* (BAM16482.1); PVX, *Potato virus X* (AAF89747.1); SsDRV, *Sclerotinia sclerotiorum debilitation-associated RNA virus* (YP_325662.1); ShVX, *Shallot virus X* (NP_620648.1); BMV, *Brome mosaic virus* (NP_041197.1); AMV, *Alfalfa mosaic virus* (YP_053235.1); GLRAV-3, *Grapevine leafroll-associated virus 3* (NP_813795.3); BYV, *Beet yellows virus* (NP_733949.1); LIYV, *Lettuce infectious yellows virus* (Q83045.2); GLRAV-7, *Grapevine leafroll-associated virus 7* (YP_004935919.1); HCV, *Hepatitis C virus* (ALB38672.1); BVDV-1, *Bovine viral diarrhea virus 1* (BAC55961.1); JEV, *Japanese encephalitis virus* (AEV57608.1); HEV, *Hepatitis E virus* (ALM55661.1); AHEV, *Avian hepatitis E virus* (AAL13366.1); SJNNV, *Striped Jack nervous necrosis virus* (NP_599247.1); RGNNV, *Redspotted grouper nervous necrosis virus* (ACV07658.1); SINV, *Sindbis virus* (AGT56188.1); VEEV, *Venezuelan equine encephalitis virus* (AAM28636.1); TBSV, *Tomato bushy stunt virus* (ACT67403.1); TNV-A, *Tobacco necrosis virus A* (ADE10194.1); TMV, *Tobacco mosaic virus* (AIL54434.1); TRV, *Tobacco rattle virus* (AHG52750.1); BSMV, *Barley stripe mosaic virus* (NP_604481.1); RsEV-3, *Rhizoctonia solani endornavirus 3* (KX349065); RsFV-1, *Rhizoctonia solani flexivirus 1* (KX349064); RsFV-2, *Rhizoctonia solani flexivirus 2* (KX349069), RsRV-1, *Rhizoctonia solani RNA virus 1* (KX349068); RsRV-2, *Rhizoctonia solani RNA virus 2* (KX349067); RsRV-3, *Rhizoctonia solani RNA virus 3* (KX349066).

The order *Tymovirales* comprises four viral families. Viruses belonging to the *Alphaflexiviridae*, *Betaflexiviridae* and *Gammaflexiviridae* feature a filamentous morphology, whereas members of the *Tymoviridae* are icosahedral. So far, three mycoviruses belonging to the order *Tymovirales* have been recognized by the ICTV; all of them represent own genera [8]. Whereas *Botrytis virus X* (*Botrexvirus*) and S*clerotinia sclerotiorum debilitation-associated RNA virus* (*Sclerodarnavirus*) belong to the *Alphaflexiviridae*, *Botrytis virus F* represents the only member of the family *Gammaflexiviridae* [54–56]. The search for conserved RdRp domains with the deduced gene products of the virome of *R*. *solani* DC17 revealed the presence of two domains assigned to pfam00978, which were distantly related to each other and to several members of the order *Tymovirales*. Sequence comparisons with the type species of all genera within the order allowed the identification of the seven conserved motifs A to G (Fig 5). A phylogenetic analysis of this region including all families of the alphavirus-like super family, placed both viruses within the order *Tymovirales* (Fig 6). They form a distinct clade, which indicates that they may represent members of a so far uncharacterized family within this order. The novel viruses were named *Rhizoctonia solani flexivirus 1* (RsFV-1) and *Rhizoctonia solani flexivirus 2* (RSFV-2).

### *Sclerotinia sclerotiorum RNA virus L* -like viruses in *R*. *solani* DC17

**Fig 7:**
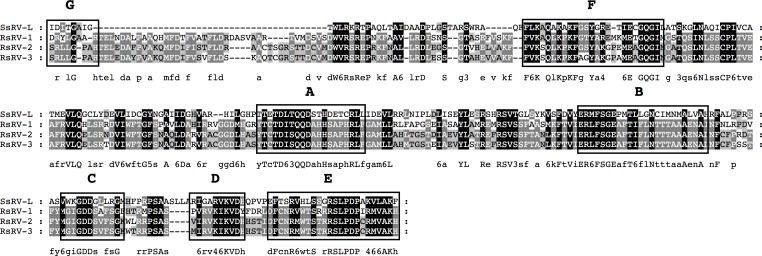
Identification of the conserved motifs A to G in the RdRp domain of a novel group of mycoviruses related to SsRV-L. The RdRp domains of the three newly identified mycoviruses *Rhizoctonia solani RNA virus 1 to 3* (RsRV-1, -2, -3) and *Sclerotinia sclerotiorum RNA virus L* (SsRV-L) (ACE88957.1) were aligned using MEGA 6.06 and the sequence alignment algorithm MUSCLE [45]. Conserved motifs A to G were marked according to Bruenn 2003, Černý et al. 2014, Koonin et al. 1993, Boehr et al. 2014 and Xu et al. 2003 [30, 42–44]. Shading indicates level of conservation and the consensus sequence is displayed.

The search for conserved RdRp domains in the *R*. *solani* DC17 virome also revealed the presence of three domains, representing the pfam00978 group that were closely rated to each other, but showed no clear affiliation to any of the recognized families of the alphavirus-like superfamily. Instead, they display similarities to the so far unassigned mycovirus *Sclerotinia sclerotiorum RNA virus L* (SsRV-L), as indicated by BLAST analysis. SsRV-L is a positive-strand RNA virus of 6,043 nts, which carries one large ORF of 1965 aa featuring conserved domains for a methyltransferase, helicase and RdRp [57]. Multiple alignments with other conserved RdRp domains of pfam00978, allowed the identification of the seven conserved motifs A to G (Fig 7). A phylogenetic analysis of this region including different type species from families of the alphavirus-like superfamily showed that the three viruses found in *R*. *solani* DC17 form a separate clade together with SsRV-L. This group shows a distant relationship to the families of the *Hepeviridae* and *Alphatetraviridae* (Fig 6). Two of the three RdRp domains within this group share a very high sequence similarity of 93% (Table 1), indicating that they possibly belong to the same species. However, comparison of the putative replicase protein spanning the conserved motifs of the methyltransferase, helicase and RdRp reduced this similarity to 77.6% on the amino acid level and to 68.8% on the nucleotide level. Appling the species demarcation criteria of the next suitable relative, the *Tymovirales* (sequence homology of the RdRp gene < 80% on amino acid or < 72% on nucleotide level) [8], the two RdRp domains can be considered as belonging to different viral species. For this reason, the identified viruses were named *Rhizoctonia solani RNA virus 1* (RsRV-1), *Rhizoctonia solani RNA virus 2* (RsRV-2), *Rhizoctonia solani RNA virus 3* (RsRV-3).

**Table 1:** Similarity comparison between *Rhizoctonia solani RNA virus 1* (RsRV-1), *Rhizoctonia solani RNA virus 2* (RsRV-2), *Rhizoctonia solani RNA virus 3* (RsRV-3) and *Sclerotinia sclerotiorum RNA virus L* (SsRV-L) using an 810 bp fragment (nt) and the corresponding amino acid sequence (aa) of the viral RdRps spanning the conserved motifs A to G.

Similarity on nt level \ Similarity on aa level [%]	SsRV-L	RsRV-1	RsRV-2	RsRV-3
SsRV-L		46.1	46.0	45.2
RsRV-1	34.8		78.9	63.4
RsRV-2	34.1	93.2		66.1
RsRV-3	33.0	70.8	71.1	

### Putative member of the family *Partitiviridae* identified in *R*. *solani* DC17

**Fig 8:**
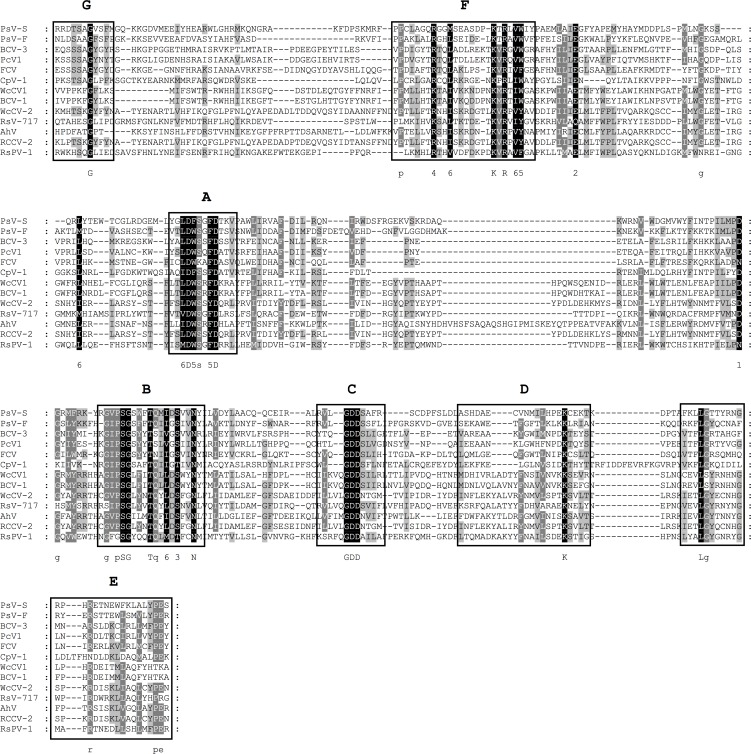
Identification of the conserved motifs A to G in the RdRp domain of the family *Partitiviridae*. The RdRp domains of the newly identified *Rhizoctonia solani partitivirus 1* (RsPV-1) (KX349061) and recognized species within the *Partitiviridae*, including the type species, [8] were aligned using MEGA 6.06 and the sequence alignment algorithm MUSCLE [45]. Conserved motifs A to G were marked according to Bruenn 2003, Černý et al. 2014, Koonin et al. 1993, Boehr et al. 2014 and Xu et al. 2003 [30, 34, 42–44]. PsV-S, *Penicillium stoloniferum virus S* (YP_052856.2); PsV-F, *Penicillium stoloniferum virus F* (YP_271922.1); BCV-3, *Beet cryptic virus 3* (AAB27624.1); PcV1, *Pepper cryptic virus 1* (AEJ07890.1); FCV, *Fig cryptic virus* (YP_004429258.1); CpV-1, *Cryptosporidium parvum virus 1* (BAU19336.1); WcCV1, *White clover cryptic virus 1* (YP_086754.1); BCV-1, *Beet cryptic virus 1* (YP_002308574.1); WcCV-2, *White clover cryptic virus 2* (YP_007889821.1); AhV RCCV-2, *Red clover cryptic virus 2* (YP_007889823.1); RsV-717, *Rhizoctonia solani virus 717* (AJE29742.1). Shading indicates level of conservation and the consensus sequence is displayed.

**Fig 9:**
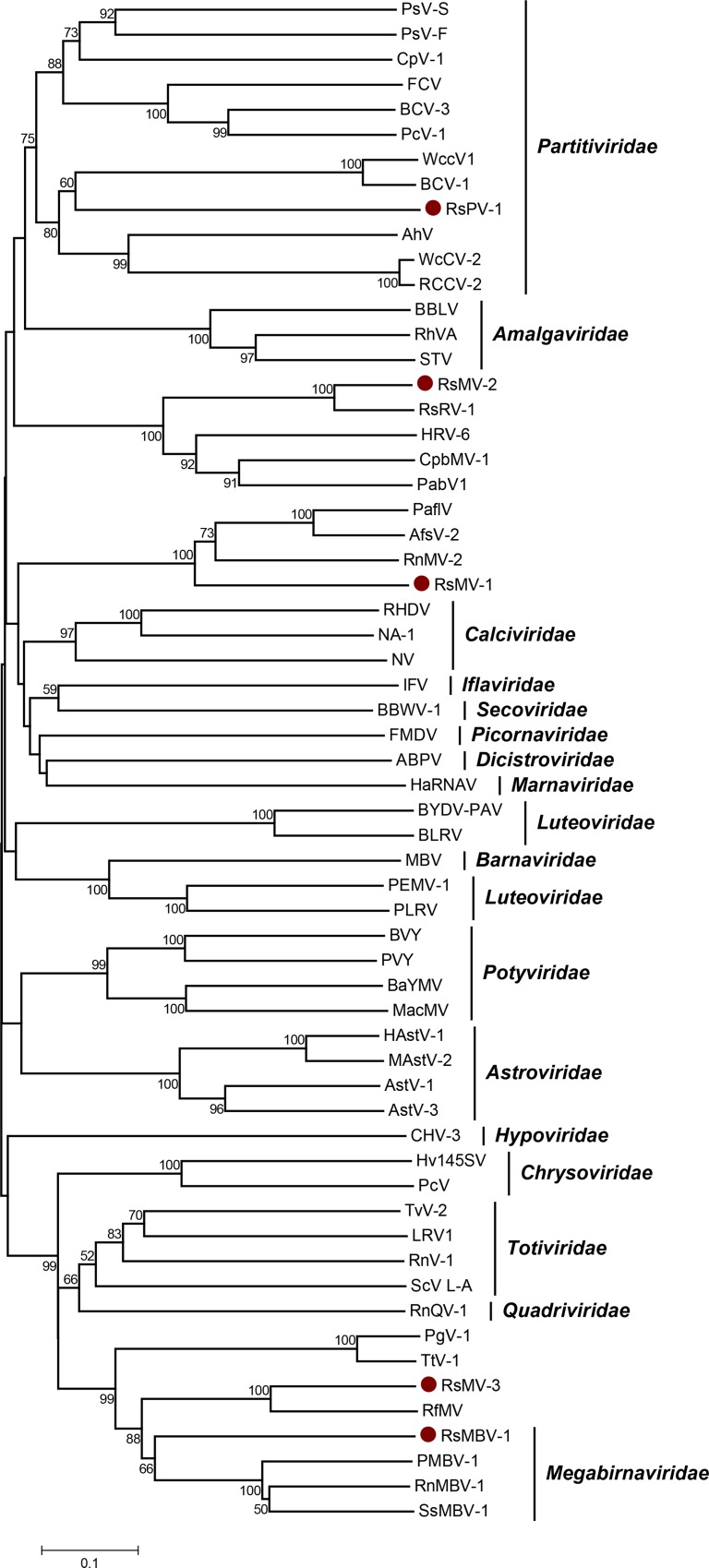
Phylogenetic analysis of selected type species within the families of the picornavirus-like superfamily. An unrooted phylogenetic tree of the picornavirus-like superfamily was calculated based on the alignment of the RdRp domain spanning the conserved motifs A to G using the neighbor-joining method with 1000 bootstrap replicates. The scale represents a genetic distance of 0.1 amino acid substitutions per site. Bootstrap values > 50% are not shown. Newly identified species are marked with dots and compared to their next relatives and selected type species within families proposed to form the picornavirus-like superfamily [8, 29]. Analysis was conducted using MEGA 6.06 and the sequence alignment algorithm MUSCLE [45]. PMBV-1, *Pleosporales megabirnavirus 1* (ALO50147.1); RnMBV-1, Rosellinia necatrix megabirnavirus 1/W779 (YP_003288763.1); SsMBV-1, *Sclerotinia sclerotiorum megabirnavirus 1* (YP_009143529.1); TtV-1, *Thelephora terrestris virus 1* (YP_009209482.1); RfV-1, *Rhizoctonia fumigatavirus* 1 (AJE29745.1); PgV-1, *Phlebiopsis gigantea mycovirus dsRNA 1* (YP_003541123.1); GaRV-6, *Gremmeniella abietina RNA virus 6* (AIU98624.1); HRV-6, *Heterobasidion RNA virus 6* (AHA82547.1); FgV-4, *Fusarium graminearum dsRNA mycovirus 4* (YP_003288790.1); PabV-1, *Penicillium aurantiogriseum bipartite virus 1* (YP_009182335.1); CpbMV-1, *Cryphonectria parasitica bipartite mycovirus 1* (YP_007985675.1); RsRV-1, *Rhizoctonia solani dsRNA virus 1* (AFZ85210.1); AfsV-2, *Aspergillus foetidus slow virus 2* (CCD33025.1), PaflV, *Penicillium aurantiogriseum foetidus-like virus* (YP_009182156.1); RnMV-2, *Rosellinia necatrix mycovirus 2* (BAM36407.1); PsV-F, *Penicillium stoloniferum virus F* (YP_271922.1); BCV-3, *Beet cryptic virus 3* (AAB27624.1); PcV1, *Pepper cryptic virus 1* (AEJ07890.1); FCV, *Fig cryptic virus* (YP_004429258.1); CpV-1, *Cryptosporidium parvum virus 1* (BAU19336.1); WcCV1, *White clover cryptic virus 1* (YP_086754.1); BCV-1, *Beet cryptic virus 1* (YP_002308574.1); WcCV-2, *White clover cryptic virus 2* (YP_007889821.1); AhV RCCV-2, *Red clover cryptic virus 2* (YP_007889823.1); RsV-717, *Rhizoctonia solani virus 717* (AJE29742.1); BBLV, *Blueberry latent virus (*YP_003934623.1); RhVA, *Rhododendron virus A* (ADM36020.1); STV, *Southern tomato virus* (ALM54938.1); AstV-1, *Avastrovirus 1* (CAB95006.3); AstV-3, *Avastrovirus 3* (AAF60952.1); HAstV-1, *Human astrovirus 1* (Q67726.1); MAstV-2, *Mamastrovirus 2* (AII82242.1); MBV, *Mushroom bacilliform virus* (NP_042510.2); NAV-1, *Newbury agent 1* (YP_529897.1); NV, *Norwalk virus* (AAC64602.1); RHDV, *Rabbit hemorrhagic disease virus* (AMQ25852.1); Hv145SV, *Helminthosporium victoriae 145S virus* (YP_052858.1); PcV, *Penicillium chrysogenum virus* (YP_392482.1); ABPV, *Acute bee paralysis virus* (Q9DSN9.1); CHV-3, *Cryphonectria hypovirus 3* (NP_613266.1); IFV, *Infectious flacherie virus* (ADP24157.1); BYDV-PAV, *Barley yellow dwarf virus-PAV* (CAH18868.1); BLRV, *Bean leafroll virus* (ALX34940.1); PEMV-1, *Pea enation mosaic virus 1* (P29154.2); PLRV, *Potato leafroll virus* (AHA43772.1); HaRNAV, *Heterosigma akashiwo RNA virus* (NP_944776.1); FMDV, *Foot-and-mouth disease virus—type O* (BAU20293.1); BaYMV, *Barley yellow mosaic virus* (BAG70349.1); BVY, *Blackberry virus Y* (YP_851006.1); MacMV, *Maclura mosaic virus* (AAB02823.1); PVY, *Potato virus Y* (BAN16607.1); RnQV1, *Rosellinia necatrix quadrivirus 1* (BAM93353.1); BBWV-1, *Broad bean wilt virus 1* (AAX12375.1); GLV, *Giardia lamblia virus* (NP_620070.1); LRV-1, *Leishmania RNA virus 1* (AHJ90430.1); ScV-L-A, *Saccharomyces cerevisiae virus L-A* (NP_620495.1); TVV-2, *Trichomonas vaginalis virus 2* (AKE98370.1), RsPV-1, *Rhizoctonia solani partitivirus 1* (KX349061); RsMV-1, *Rhizoctonia solani mycovirus 1* (KX349063); RsMV-2, *Rhizoctonia solani mycovirus 2* (KX349062); RsMV-3, *Rhizoctonia solani mycovirus 3* (KX349070); RsMBV-1, *Rhizoctonia solani megabirnavirus 1* (KX349071).

The virome analysis revealed the presence of one contig of 1958 nt, which codes for a putative protein of 633 aa. The protein contains a conserved RdRp domain showing the highest sequence similarity of 56% to the conserved RdRp domain of *Rosellinia necatrix partitivirus 7*. An alignment with several recognized members of the family *Partitiviridae* allowed the identification of the conserved domains A to F. It is reported that motif G is not well conserved within this viral family [30]. Nevertheless, an alignment with several other members of the picornavirus-like superfamily indicated the presence of a conserved glycine residue at the N-terminal region of the F motif which we considered as motif G (Fig 8). A phylogenetic analysis of the region spanning these motifs classified this virus as a member the genus *Alphapartitivirus* (Fig 9). A second contig of 1805 nt carried an ORF coding for a putative protein of 586 aa, which shows highest sequence similarity (24.7%) to the coat protein of *Sclerotinia sclerotiorum partitivirus S*. To date, three different members of the *Partitiviridae* have been characterized in the genus *Rhizoctonia*. Two of them, *Rhizoctonia solani dsRNA virus 2* and *Rhizoctonia solani virus 717*, were isolated from *R*. *solani*. The putative partitivirus identified in this study, showed only low sequence similarity of less than 30% within the conserved region of the RdRp to these viruses. It is therefore most likely that this novel virus, referred to *Rhizoctonia solani partitivirus 1* (RsPV 1), is a new member of the genus *Alphapartitivirus*.

### *Aspergillus foetidus slow virus 2* -like virus in *R*. *solani* DC17

**Fig 10:**
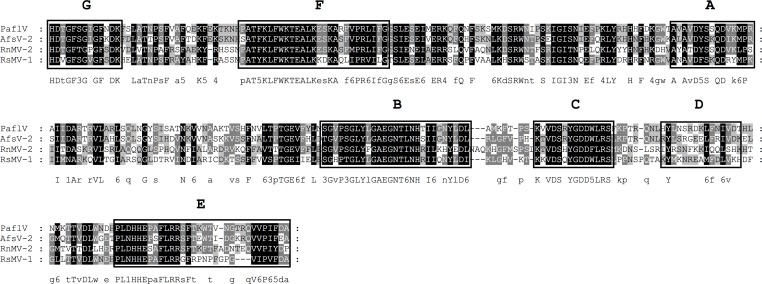
Identification of the conserved motifs A to G in the RdRp domain of *Rhizoctonia solani mycovirus-1* (RsMV-1). The RdRp domains of the newly identified *Rhizoctonia solani mycovirus 1* (RsMV-1) (KX349063) and *Aspergillus foetidus slow virus 2* (AfsV-2) (CCD33025.1), *Penicillium aurantiogriseum foetidus-like virus* (PaflV) (YP_009182156.1) and *Rosellinia necatrix mycovirus 2* (RnMV-2) (BAM36407.1) were aligned using MEGA 6.06 and the sequence alignment algorithm MUSCLE [45]. Conserved motifs A to G were marked according to Bruenn 2003, Černý et al. 2014, Koonin et al. 1993, Boehr et al. 2014 and Xu et al. 2003 [30, 34, 42–43]. Shading indicates level of conservation and the consensus sequence is displayed.

The search for conserved RdRp domains in the *R*. *solani* DC17 virome showed the presence of a domain belonging to the cd01699 group. Different members of the picornavirus-like superfamily, like the *Claciviridae* or the *Partitiviridae*, share this group of domain. A multiple alignment with different members of the picornavirus-like superfamily allowed the identification of the conserved motifs A to G (Fig 10). A BLAST analysis of the region revealed that the RdRp domain shows significant sequence similarities to three so far unclassified viruses. The closest relative with 54% sequence identity is *Aspergillus foetidus slow virus 2* (AfsV-2). The virus present in *R*. *solani* DC17 was named *Rhizoctonia solani mycovirus 1* (RsMV-1). The phylogenetic analysis grouped RsMV-1 with AfsV-2, *Penicillium aurantiogriseum foetidus-like virus* (PaflV) and *Rosellinia necatrix mycovirus 2* (RnMV-2) into one clade, which is distantly related to the *Calciviridae* (Fig 9). The contig, carrying the RdRp domain of RsMV-1, is 4,130 nt in size and encodes putative protein is 1376 aa. The conserved RdRp domain is positioned within the N-terminal region of the protein. A similar organization is reported for RnMV-2, also referred to as *yado-kari virus 1* [58], whereas AfsV-2 has a smaller genome and the conserved RdRp domain is positioned in the center of the protein [59]. Both, RnMV-2 and AfsV-2 are described to be unique viruses which hijack the capsid protein of an unrelated dsRNA virus [58, 59]. Whether this unusual behavior also holds true for RsMV-1 needs further investigation.

### *Rhizoctonia solani dsRNA virus 1* -like virus in *R*. *solani* DC17

**Fig 11:**
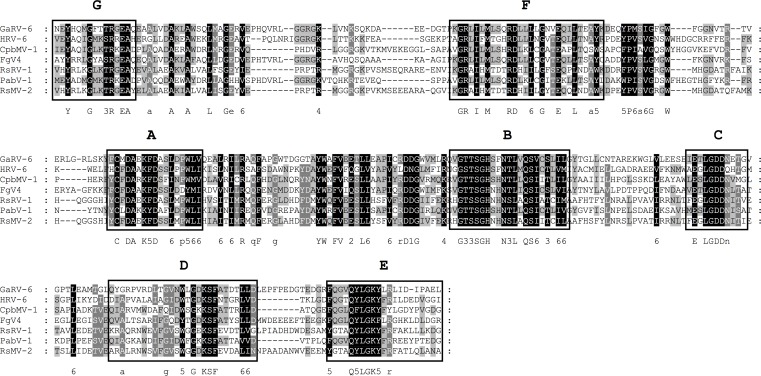
Identification of the conserved motifs A to G in the RdRp domain of *Rhizoctonia solani mycovirus-2* (RsMV-2). The RdRp domains of the newly identified *Rhizoctonia solani mycovirus 2* (RsMV-2) (KX349062) and the unassigned mycoviruses *Gremmeniella abietina RNA virus 6* (GaRV-6) (AIU98624.1); *Heterobasidion RNA virus 6* (HRV-6) (AHA82547.1); *Fusarium graminearum dsRNA mycovirus 4* (FgV-4) (YP_003288790.1); *Penicillium aurantiogriseum bipartite virus 1* (PabV-1) (YP_009182335.1); *Cryphonectria parasitica bipartite mycovirus 1* (CpbMV-1) (YP_007985675.1) and *Rhizoctonia solani dsRNA virus 1* (RsRV-1) (AFZ85210.1) were aligned using MEGA 6.06 and the sequence alignment algorithm MUSCLE [46]. Conserved motifs A to G were marked according to Bruenn 2003, Černý et al. 2014, Koonin et al. 1993, Boehr et al. 2014 and Xu et al. 2003 [30, 34, 42–44]. Shading indicates level of conservation and the consensus sequence is displayed.

*Rhizoctonia solani dsRNA virus 1* (RsRV-1) is an unclassified bi-segmented dsRNA virus, which was isolated from *R*. *solani* AG-1 IA [23]. Its genome structure is similar to those of the *Partitiviridae* and both share a RdRp of pfam00680 group. Nevertheless, a phylogenetic analysis indicates that it is only distantly related to the family *Partitiviridae* [23]. The BLAST analysis of the multiple RdRps, isolated from *R*. *solani* DC17 showed that one of the RdRps is very similar to the one of RsRV-1 carrying the same conserved motifs A to G (Fig 11). Although, both viruses share 81.6% identity within the conserved RdRp domain, the comparison of the whole replicase indicated a lower similarity level of 76.8%.

Thus, this virus found in *R*. *solani* DC17, is novel and was named *Rhizoctonia solani mycovirus 2* (RsMV-2). Both viruses are closely related, which was also confirmed by the phylogenetic analysis of the RdRp domain. Both viruses cluster together and form a distinct clade together with some so far unclassified viruses like *Heterobasidion RNA virus 6* or *Curvularia thermal tolerance virus* (Fig 9).

### Putative members of the family *Megabirnaviridae* in *R*. *solani* DC17

**Fig 12:**
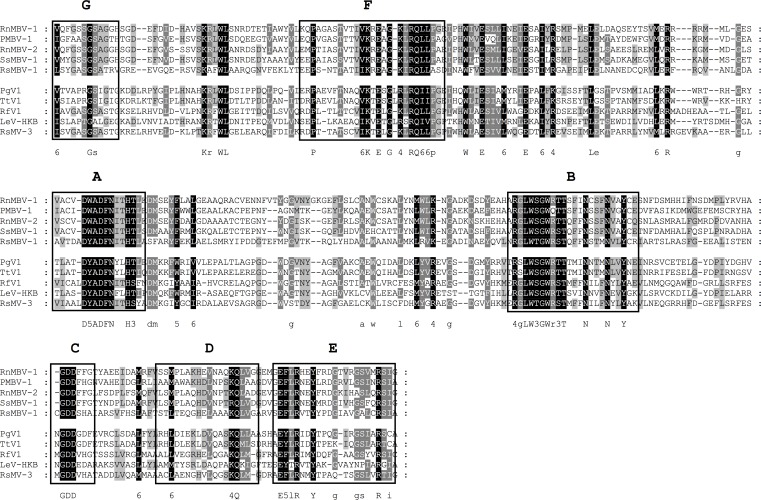
Identification of the conserved motifs A to G in the RdRp domain of *Rhizoctonia solani megabirnavirus 1* (RsMBV-1) and *Rhizoctonia solani mycovirus 3* (RsMV-3). The RdRp domains of the newly identified *Rhizoctonia solani megabirnavirus 1* (RsMBV-1) (KX349071), *Rhizoctonia solani mycovirus 3* (RsMV-3) (KX349070) and proposed members of the family *Megabirnaviridae* were aligned using MEGA 6.06 and the sequence alignment algorithm MUSCLE [45]. Conserved motifs A to G were marked according to Bruenn 2003, Černý et al. 2014, Koonin et al. 1993, Boehr et al. 2014 and Xu et al. 2003 [30, 34, 42–44]. PMBV-1, *Pleosporales megabirnavirus 1* (ALO50147.1); RnMBV-1, *Rosellinia necatrix megabirnavirus 1*/W779 (YP_003288763.1); RnMBV-2, *Rosellinia necatrix megabirnavirus 2-W8* (YP_009227124.1); SsMBV-1, *Sclerotinia sclerotiorum megabirnavirus 1* (YP_009143529.1); TtV-1, *Thelephora terrestris virus 1* (YP_009209482.1); RfV-1, *Rhizoctonia fumigata mycovirus* (AJE29745.1); LeV-HKB, *Lentinula edodes mycovirus HKB* (BAG71788.2); PgV-1, *Phlebiopsis gigantea mycovirus dsRNA 1* (YP_003541123.1). Shading indicates level of conservation and the consensus sequence is displayed.

Currently, *Rosellinia necatrix megabirnavirus 1* (RnMBV-1) is the only recognized member of the family *Megabirnaviridae*. It possesses a bi-segmented double-stranded RNA genome and the segments are encapsidated separately into isometric particles. The first segment carries two ORFs, which code for an RdRp of the pfam02123 protein family and a coat protein. The second segment encodes two proteins of unknown function [60, 61]. In the virome of *R*. *solani* DC17, two conserved RdRp domains of the pfam02123 group were detected. One showed significant similarity of 68% to the RdRp of RnMBV-1, whereas the second RdRp domain displayed lower similarity of only 29%. Sequence comparisons with different members of the picornavirus-like superfamily allowed the identification of the conserved motifs A to G in both novel RdRps (Fig 12). A phylogenetic analysis of this region including different type species within the picornavirus-like superfamily as well as some so far uncharacterized viruses, grouped both RdRps into one clade with the families *Totiviridae*, *Chrysoviridae*, *Quadriviridae* and *Megabirnaviridae* (Fig 9). However, the first RdRp clusters with RnMBV-1 and some other proposed members of the family *Megabirnaviridae*. The second RdRp shows close a relationship to the recently characterized RdRp domain of the unclassified mycovirus *Rhizoctonia fumigate virus 1* (RfV-1) [25]. RfV-1 is a one-segmented dsRNA virus. It is proposed to form a new genus together with *Phlebiopsis gigantean large virus-1* (PgV-1) and *Lentinula edodes mycovirus* (HKB and HKA) [25]. The analysis indicates, that the virome of *R*. *solani* DC17 contains two mycoviruses, which are related to the family *Megabirnaviridae*. The first virus seems to be a member of the genus *Megabirnavirus* and was named *Rhizoctonia solani megabirnavirus 1* (RsMBV-1). The second virus is closely related to a different group of so far unclassified viruses and was named *Rhizoctonia solani mycovirus 3* (RsMV-3). To elucidate whether RsMV-3 belongs to a new viral family or a new proposed genus within the *Megabirnaviridae* [25], further criteria apart from the phylogeny of the RdRp domain need to be considered.

### Methodological approach and its implication for virus identification

Our results demonstrate that a deep sequencing approach is the method of choice for the analysis of diverse viromes and that the characterization of the conserved RdRp domain is the best way to get a valid overview about viral diversity in such complex RNA virus viromes. However, our study also showed the two main pitfalls of virus characterization in virome samples. In general, virome analysis faces the problem, that no marker gene for targeted amplification like the ribosomal internal transcribed spacer (ITS) is present in viruses. Instead, other methods for the enrichment of viral sequences are used, which all bring along certain disadvantages. The most popular method is the purification of virus particles, which seems natural, as the viruses used to be defined as capsid-coding organisms [47]. However, the identification of the capsidless hypoviruses and their acceptance by the ICTV changed this [46]. Further progress in the field of mycovirus research revealed that the absence of a coat protein is a common feature among mycoviruses making the approach of particle purification rather unsuitable for virome analysis in fungi. An alternative approach recently used for the characterization of mycoviruses, utilized siRNAs from infected fungi [62]. Even though they were able to detect mycoviruses belonging to the *Narnaviridae* (ssRNA, unencapsidated), as well as to the *Partitiviridae* (dsRNA, encapsidated), it is not sure if all mycoviruses are delectable by this method, as interference with RNA silencing seems to be an important trait in mycoviruses [63]. The sequencing of total RNA and DNA is a further popular method but viruses of low a titer might not be detected [63]. Especially in virome analysis of environmental samples, the first level assignment of reads belonging to novel viruses is another problem, when no separation between viral and host sequences has been applied [64]. To overcome the limitations described, we used an extraction of dsRNA, which is a replicative intermediate of all RNA viruses, as starting material for library preparation in order to maximize the number of reads derived from viruses. Still, the coverage between contigs differed enormously, most likely due to differences in the virus titer within the sample, as the whole dsRNA extract was used for analysis, compared to individual dsRNA band excision from the gel. Using equimolar ratios of purified dsRNA fragments would probably improve the uniformity in coverage, but may cause the problem of missing certain species, which, due to low virus titers, are not visualized by gel electrophoresis. However, the coverage can be used for an indication of the dominance of certain viral species in the virome, which is especially interesting, as hypovirulence is reported to be correlated to higher virus titers [65, 66]. Following this hypothesis, RsMV 17 could be of special interest, as its coverage was 17 times higher than the average. Still, some of the contigs representing novel species, like RsFV-1 and RsFV-2 displayed a very low coverage indicating that the contigs do not represent full-length genome components.

The second problem is the limited number of characterized mycoviral sequences, which can be used as reference for species assignment of the generated contigs. Apart from conserved regions, sequence similarities are too low to draw conclusions about the phylogeny of the virus [67]. The RdRp domain is the best available indicator to identify also novel viruses, sharing no other homologies with known viruses and represents the key for the reconstruction of evolutionary relationships between those viruses [29–31]. In general, virus taxonomy is a challenging field since high mutation rates and frequent exchange of whole gene modules lead to a very high sequence divergence, which makes reasonable alignments virtually impossible [67]. Nevertheless, the analysis the RdRp allows clustering above the order level in so-called superfamilies. The identification of the conserved motifs within the RdRp domain is an important point, since only the comparison of homologous sequences will lead to a valid taxonomic assignment [67]. We identified the conserved motifs A to G of the novel viruses, as well as in reference sequences derived from the NCBI database and used an alignment of this region for a first taxonomic classification.

This approach enabled us to get a first and valid impression of the viral diversity of the species inhabiting *R*. *solani* DC17 and allowed an identification of novel viruses which might be of special interest in the fields of biocontrol, virus biology and taxonomy. For example, the analysis revealed the presence of the novel mitovirus RsMV-20, which is closely related to *Thanatephorus cucumeris mitovirus* (TcMV). An infection of *Rhizoctonia* with TcMV resulted in decreased virulence of the host [21, 68]. Studies using this hypovirulent isolate of *Rhizoctonia* (AG-3), infected with TcMV, demonstrated its potential use as biocontrol agent [6]. This makes RsMV-20 an interesting candidate for further analysis in regard to hypovirulence induction. Also, a further characterization of RsMBV-1 is promising, since, so far, all described megabirnaviruses, apart from *Pleosporales megabirnavirus 1*, which was detected in the virome of marine fungi, were associated with hypovirulence of their hosts [61, 69–71]. As well, the identification of RsMV-1, which is closely related to AfV-S2 and RnMV-2 is an interesting finding. AfV-S2 and RnMV-2 are reported to feature a rather unusual lifestyle since they possess a one-segmented ssRNA genome, which hijacks the capsid protein (CP) of an unrelated dsRNA virus [57, 58]. The coat protein of RnMV-2 originates from *Rosellinia necatrix mycovirus 1*, which belongs to a group of novel viruses distantly related to the *Totiviridae* [57]. In case of AfV-S2, the capsid protein is derived from a member of the *Victoriviruses* (*Totiviridae)* [58]. The close relationship between those viruses and the newly identified virus RsMV-1 raises the hypothesis that also RsMV-1 might hijack a CP of another virus. As no totivirus or close relative is present in the virome of *R*. *solani* DC17, RsMV-1 would have to use the CP of another virus, like *Rhizoctonia solani partitivirus-1* or *Rhizoctonia solani megabirnavirus-1*. Either way, a finding like this would be interesting in regard to virus interactions and evolution.

Still, for a full-value assignment into viral families and a biological characterization of the novel viruses, more criteria than the phylogeny of the RdRp have to be analyzed, especially when the viruses belong to novel viral groups. The next steps need to be the determination of full-length sequences and the isolation of single virus species to study their biological effects by e.g. purification and transfection of viral particles, the construction of infectious full-length cDNA-clones, or the elimination of certain virus by mycelial fragmentation, hyphal tipping or protoplastation [72–75].

## Conclusion

This is the first study characterizing a complex virome from a hypovirulent isolate of *R solani* to explore the diversity of mycoviruses in this fungus. By the characterization of the conserved RdRp domains, we identified 17 different mycoviruses of which 16 domains represent novel species not characterized before. To our knowledge no comparable number of virus species belonging to at least eight different families, has been previously reported to infect a single fungal isolate. The research conducted here is a step forward in exploring the mycoviruses of *R*. *solani*. The enormous diversity identified in this study raises questions about the interactions of viruses as well as the dynamics of viral communities in fungi. More detailed analysis of the hypovirulent isolate DC17 and its inhabiting viruses might help to get insights into this still unexplored field of mycovirus research. Work, like the one presented here, will help to further populate public databases with viral sequences, which in turn will help to solve difficulties in virus identification and classification in the future [64].

## Supporting Information

S1 Table Primer sequences used for reamplification of fragments encoding the viral RdRp domains identified in the virome of *R*. *solani* DC17. (DOCX)
