# Supplementary material for: Deep Sequencing Analysis Reveals the Mycoviral Diversity of the Virome of an Avirulent Isolate of Rhizoctonia solani AG-2-2 IV
Source: PLoS One. 2016 Nov 4;11(11):e0165965. doi: 10.1371/journal.pone.0165965 (PMC5096721; doi:10.1371/journal.pone.0165965)
Supplement: S1 Table — (DOCX) [file pone.0165965.s001.docx]

**Supporting Information**

**S1 Table.** Primer sequences used for reamplification of fragments encoding the viral RdRp domains identified in the virome of *R. solani* DC17.
